# Novel and nodulation-regulated microRNAs in soybean roots

**DOI:** 10.1186/1471-2164-9-160

**Published:** 2008-04-10

**Authors:** Senthil Subramanian, Yan Fu, Ramanjulu Sunkar, W Brad Barbazuk, Jian-Kang Zhu, Oliver Yu

**Affiliations:** 1 Donald Danforth Plant Science Center, 975 N Warson Road, St Louis, MO, 63132, USA; 2 Department of Biochemistry and Molecular Biology, Oklahoma State University, Stillwater, OK, 74078, USA; 3 Center for Plant Cell Biology, Department of Botany and Plant Sciences, University of California, Riverside, CA, 92521, USA

## Abstract

**Background:**

Small RNAs regulate a number of developmental processes in plants and animals. However, the role of small RNAs in legume-rhizobial symbiosis is largely unexplored. Symbiosis between legumes (e.g. soybean) and rhizobia bacteria (e.g. *Bradyrhizobium japonicum*) results in root nodules where the majority of biological nitrogen fixation occurs. We sought to identify microRNAs (miRNAs) regulated during soybean-*B. japonicum *symbiosis.

**Results:**

We sequenced ~350000 small RNAs from soybean roots inoculated with *B. japonicum *and identified conserved miRNAs based on similarity to miRNAs known in other plant species and new miRNAs based on potential hairpin-forming precursors within soybean EST and shotgun genomic sequences. These bioinformatics analyses identified 55 families of miRNAs of which 35 were novel. A subset of these miRNAs were validated by Northern analysis and miRNAs differentially responding to *B. japonicum *inoculation were identified. We also identified putative target genes of the identified miRNAs and verified *in vivo *cleavage of a subset of these targets by 5'-RACE analysis. Using conserved miRNAs as internal control, we estimated that our analysis identified ~50% of miRNAs in soybean roots.

**Conclusion:**

Construction and analysis of a small RNA library led to the identification of 20 conserved and 35 novel miRNA families in soybean. The availability of complete and assembled genome sequence information will enable identification of many other miRNAs. The conserved miRNA loci and novel miRNAs identified in this study enable investigation of the role of miRNAs in rhizobial symbiosis.

## Background

Symbiotic association between leguminous plants and rhizobia bacteria results in specialized nitrogen-fixing structures called root nodules. The interaction between the symbiotic partners starts with the exchange of chemical signals. Legumes release specific flavonoids (a group of small phenolic compounds) as signal molecules into the soil through root exudates. Compatible rhizobia bacteria (*Bradyrhizobium japonicum *in case of soybean) respond by producing specific lipochitooligosaccharide (LCO) bacterial signals which are in turn recognized by plants [1-3] resulting in the attachment of bacterial cells to plant root hairs. Signal transduction leading to the process of nodule development commences upon recognition of compatible bacterial LCOs on the root surface by the plants. The immediate responses are ion fluxes (Ca^2+ ^influx and Cl^- ^and K^+ ^efflux) leading to alkanization of the cytoplasm [4] and within hours, the root hairs are deformed and transcription of nodulation-specific genes begins in the root cells. Cells within the pericycle and cortical layers of the root initiate processes for cell division by ~24 h after LCO perception. By 48 h, the root hairs curl tightly to entrap the bacteria and subsequently transport them to deeper cell layers of the root via structures termed infection threads. Bacteria colonize nodule cells, differentiate into membrane enclosed bacteroids and a mature nitrogen-fixing nodule forms in 2–3 weeks period [5-7]. The physiological and cytological events during nodule development have been well-characterized. In addition, a few receptors, signaling intermediates and transcription factors involved in nodulation as well as transcripts expressed differentially during nodule development have been identified (reviewed by [8,9]). However, knowledge on the role of microRNAs (miRNAs) during nodule development is lacking. miRNAs are short ~21 nt molecules that regulate gene expression post-transcriptionally and have been identified in both animals and plants. In plants, miRNAs have been clearly shown to regulate a number of developmental and physiological processes [10-14].

Genes encoding miRNAs are complete transcriptional units and yield primary miRNAs (pri miRNA) of 70 to 300 bp in length upon transcription by RNA polymerase II. In animals, the pri miRNA is integrated in to a multiprotein complex consisting of the RNaseIII-like protein Drosha and its partner protein Pasha. Cleavage of the pri miRNA by this protein complex, results in a ~70 bp long fold back structure termed pre miRNA which is subsequently exported to the cytoplasm. There, it is cleaved by another RNase III-like enzyme called Dicer to yield a double-stranded miRNA duplex with typical 2 nt long 3' overhangs. In plants, the Dicer homolog is thought to possess both of these cleavage activities. The miRNA duplex consists of the mature miRNA and its near complementary miRNA* strand. This duplex associates with an ARGONAUTE (AGO) protein leading to simultaneous unwinding after which the mature miRNA guides AGO to complementary target mRNAs resulting in silencing of the target mRNA. The mechanism by which miRNAs regulate the abundance of their target(s) also differs between animals and plants. In animals, the complementarity between miRNAs and their mRNA targets is not very high and the repression of gene expression is caused primarily by the blockage of translation. In contrast, there is very high complementarity between miRNAs and their target mRNAs in plants and the repression of gene expression occurs primarily through the cleavage of mRNA targets (reviewed by [12]).

Computational prediction of pri miRNAs within the genome sequences can successfully identify miRNAs. The major advantage of the method is the ability to identify miRNAs independent of their abundance or spatial and temporal expression patterns [15,16]. However, it depends on the availability of extensive and assembled genome sequence data and is subject to false-discovery. An equally effective complementary approach in identifying miRNAs is cloning and analysis of small RNA sequences [17-20]. While this method still depends on the availability of genome sequence data for authentication, it has better accuracy. Combined with the deep coverage achieved by new high through put sequencing methods, this approach may allow comparison of miRNA abundance between different samples in addition to identification of novel miRNAs [21-24].

We cloned and analyzed small RNA sequences from soybean roots inoculated with *B. japonicum *to identify conserved as well as novel miRNAs that may function in symbiotic nodule development. We used soybean roots 3 h post rhizobia inoculation in an attempt to identify miRNAs that may be early regulators of nodulation. Small RNA libraries were constructed from mock-inoculated and *B. japonicum-*inoculated soybean roots. Cloning, sequencing and analysis of small RNA libraries identified 20 conserved miRNA families and 35 novel candidate miRNA families. Of these, 14 families were verified to be genuine miRNAs by Northern expression analysis. Potential mRNA targets regulated by a subset of these miRNAs were also verified by 5' RACE analysis. Importantly, we identified and verified a number of miRNAs differentially regulated in response to *B. japonicum *inoculation in soybean roots. These miRNAs are likely involved in rhizobial symbiosis; so, their identification might help advance our understanding of this process.

## Results

### Cloning and sequencing of small RNAs from soybean

**Figure 1:**
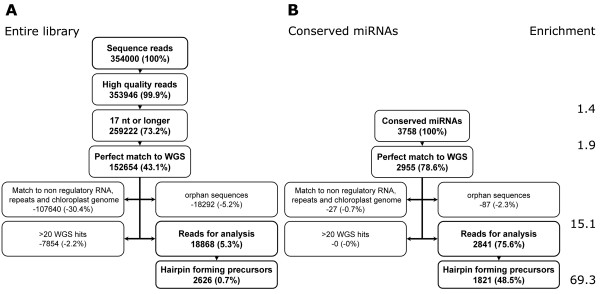
Summary of the filtering process to identify candidate miRNA sequences in the library. See text for details about each filtering process. Numbers in each box represent the read numbers and numbers in parenthesis are frequency calculated as the percentage of reads retained (or discarded) after each step of processing compared to the total number of reads. Enrichment is calculated as the percentage of conserved miRNA reads in the number of library reads retained after each step of processing.

We cloned and sequenced small RNAs from mock-inoculated (Control library) and *B. japonicum*-inoculated (3 h) soybean roots (*Bj *library). We obtained a total of 159145 sequence reads from the control library and 194855 sequence reads from the *Bj *library using deep pyrosequencing (454 Lifesciences). For computational analysis and filtering of potentially non miRNA reads, we combined reads from both the libraries. For abundance calculations, reads from Control and Bj libraries were differentiated based on ID tags. Only reads with a recognizable adapter (see Methods) sequence were retained for further analysis ('high quality reads' in Figure 1A). Since 454 sequencing is not directional, we used the 5' adapter sequence to determine the direction of each sequence read. Where necessary, the sequences were converted to their corresponding reverse complements to facilitate computational analysis. Adapter sequences were removed and the sequences were cleaned of low quality reads (see Methods). All sequences 17 nt or longer in length were retained ('17nt or longer' in Figure 1A).

### Assessment of library quality

Ideally, the length range of miRNAs in the library would be 20–24 nt while the majority of the miRNAs would be 21 nt in length [23]. However, cloning and sequencing artifacts may result in a large number of genuine miRNA sequences that deviate from this expected range. We searched the sequences from both the libraries against miRbase [25,26] to identify conserved miRNAs (see Methods for parameters used) and used the length of conserved miRNA sequences in the library as a measure of quality. The assumption is that a higher quality small RNA library will be enriched in 20–24 nt long conserved miRNAs. If a major part of the library was affected by inefficient cloning, sequencing or trimming errors, majority of the conserved miRNAs will be outside the 20–24 nt range.

**Figure 2:**
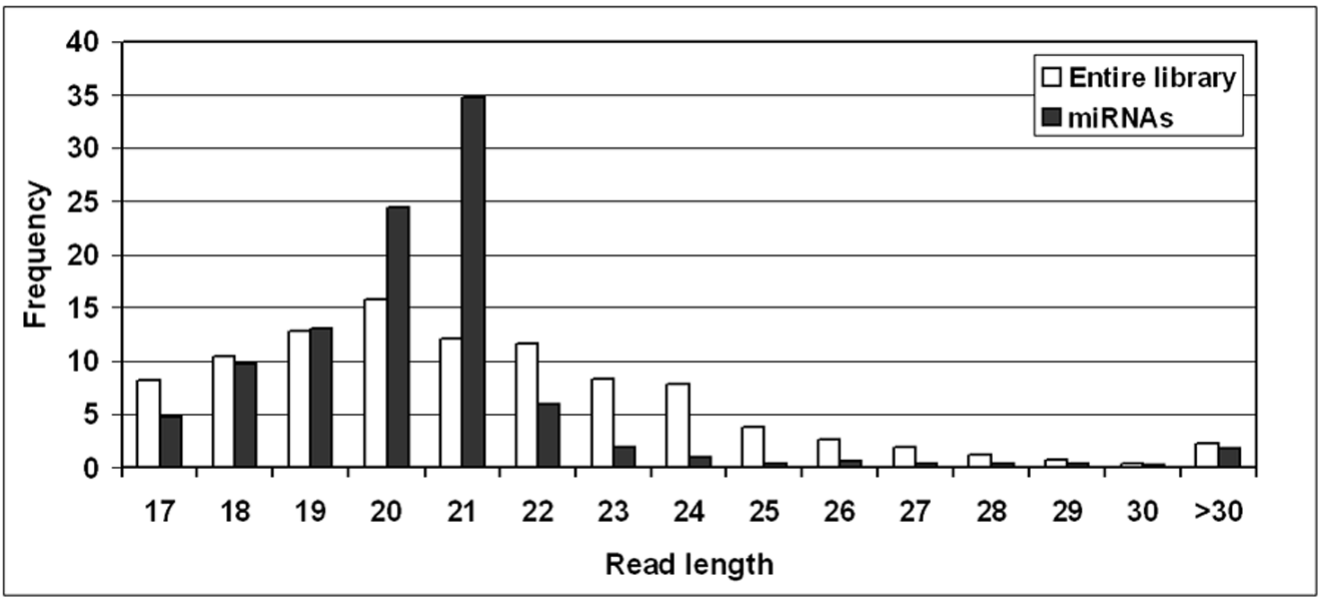
Abundance of RNA sequences of different lengths in the library. After filtering for high-quality reads and trimming, the majority of the reads in the library ranged from 17 – 31 nt in length (white bars). The majority of the conserved miRNAs identified based on homology to sequences in miRbase ranged from 20–21 nt in length suggesting that our library was of high quality (see text).

A total of 3758 sequences in our library matched previously identified miRNAs listed in miRbase ('conserved miRNAs' in Figure 1B). About 68% of these reads were 20–24 nt in length representing possibly full length mature miRNAs (Figure 2). About 28% of the reads were 17–19 nt in length. These are very likely to be genuine miRNAs shortened due to cloning, trimming and/or sequencing artifacts. Majority of the conserved miRNAs in our libraries ranged from 20–21 nt in length (~60%; Figure 2) suggesting that we had high quality small RNA libraries.

### Identifying library sequences with perfect sequence match to known soybean sequences

The best method to differentiate candidate miRNAs from other RNA sequences in the library is to identify potential hairpin forming pri miRNA precursor sequences in the genome that encompass the mature miRNA sequence [19,23]. Hence, we searched all reads in our libraries against all public soybean, genomic and expressed sequence tag (EST) sequences available (see Methods) to identify reads with perfect sequence matches to known soybean or other legume sequences. Any read that did not match a known sequence in these databases cannot be used to predict precursors and these were excluded from this stage of processing. After searching for perfect match with known soybean sequences, we retained ~43% of the reads ('Perfect match to WGS' in Figure 1A). It is expected that this would exclude some genuine miRNA sequences; therefore, we determined the number of conserved miRNA sequences excluded to estimate the loss of genuine miRNA sequences. Of the 3758 conserved miRNAs, 2955 (~79%) remained in the analysis set ('Perfect match to WGS' in Figure 1B) indicating that ~21% of genuine miRNAs were lost due to unavailability of comprehensive genome sequence information for soybean.

### Removal of non regulatory small RNA sequences and "orphan" sequences

Small RNA libraries often contain non-regulatory small RNAs including tRNAs, rRNAs, snRNAs and degradation products of protein-coding RNAs. We searched our library against several non-regulatory RNA databases (see Methods) which identified 107640 sequences from both the libraries and all of these were excluded from further analyses (Figure 1A). This excluded just one conserved miRNA (ppt-miR896; 27 reads) from the library (Figure 1B) even though it removed close to 30% of the total sequences.

To further enhance the identification of genuine miRNA sequences, we eliminated all 'orphans' (reads represented only once in both libraries combined) and retained only 'non-orphan' sequences. Differences in read length were taken into account during this process (see Methods). This clean-up process removed only ~2% of genuine miRNA sequences (Figure 1B). We also excluded library sequences that hit more than 20 individual soybean whole genome shotgun (WGS) reads since these are unlikely to be genuine miRNAs [23]. Consistently, this step did not exclude a single conserved miRNA (Figure 1B). The above described clean-up processes resulted in 4959 unique sequences (18868 reads; Figure 1A) ranging from 17 to 30 nt in length which were used for computational identification of miRNAs.

### Computational identification of miRNA sequences with potential hairpin forming precursors

Candidate miRNAs can be identified from genomic and/or EST sequences by searching for the presence of potential hairpin forming precursors encompassing the mature miRNA sequence present in the library. Using this criterium, candidate miRNAs can also be differentiated from other regulatory RNAs such as siRNAs [19,23,27]. For each candidate miRNA sequence that had a perfect match in the soybean genome, we determined the ability of sequences in the surrounding region (at least 30 bp away and not farther than 500 bp on either side) to form a hairpin structure encompassing the miRNA sequence. We identified a total of 227 unique sequences (2626 reads; Figure 1A) that had the ability to form a potential hairpin structure of which 139 were novel.

**Figure 3:**
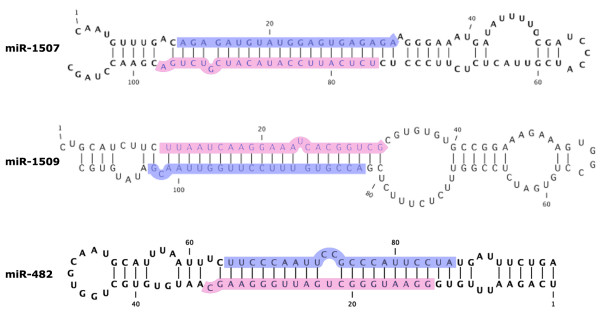
RNA secondary structure of the hairpin forming precursors of gma-MIR1507, MIR1509 and MIR482. The putative mature miRNA and miRNA* sequences identified in the library are shaded in pink and blue respectively. Nucleotide positions are numbered starting from the 5' end of the precursor sequence.

The 139 novel precursors were subjected to folding (see Methods) and these hairpin structures were manually examined if they fit the criteria (less than 8 unpaired nucleotides and no more than three consecutive unpaired nucleotides of which no more than two were asymmetrically bulged in the 25 bp stem encompassing the mature miRNA sequence) used by [23]. We selected 82 sequences that fit the criteria as candidate miRNA genes. The folded secondary structures of three of our candidates are shown in Figure 3 as examples. Finally, we clustered these sequences into 35 different families representing novel candidate miRNAs (Table 1). Of the 35 candidates, 3 had miRNA* sequences in our library (Table 1), and all three paired to their corresponding miRNAs with 2 nt 3' overhangs (Figure 3). This is strong evidence that the miRNA/miRNA* pair originated from a Dicer1-like (DCL1) processing indicating that these are genuine miRNAs [22,23]. Of the remaining 32 candidates, all of the 5 tested were validated by Northern expression analysis (Table 1, see below) suggesting that most of the novel miRNAs identified in the study could be genuine miRNAs.

**Table 1:** Novel miRNAs identified from soybean roots

**miRNA ID**	**Sequence (5'-3')^a^**	**Length**	**Validation^b^**
gma-MIR1507	UCUCAUUCCAUACAUCGUCUGA	22	mi*
gma-MIR1508	UAGAAAGGGAAAUAGCAGUU	20	N (21 nt)
gma-MIR1509	UUAAUCAAGGAAAUCACGGUCG	22	mi*, N (23 nt)
gma-MIR1510	UGUUGUUUUACCUAUUCCACC	21	N (21 nt)
gma-MIR1511	AACCAGGCUCUGAUACCAUG	20	--
gma-MIR1512	UAACUGAAAAUUCUUAAAGUA	21	--
gma-MIR1513	UGAGAGAAAGCCAUGACUUAC	21	N (21 nt)
gma-MIR1514a	UUCAUUUUUAAAAUAGGCAUU	21	--
gma-MIR1514b	UUCAUUUUUAAAAUAGACAU	20	--
gma-MIR1515	UCAUUUUGCGUGCAAUGAUCU	21	--
gma-MIR1516	CAAAAGAGCUUAUGGCUUGUA	23	--
gma-MIR1517	AGUCUUGGUCAAUGUCGUUCGAAA	24	--
gma-MIR1518	UGUGUUGUAAAGUGAAUAUCA	21	--
gma-MIR1519	UAAGUGUUGCAAAAUAGUCAUU	22	--
gma-MIR1520a^c^	UAGAACAUGAUACAUGACAGUCA	23	--
gma-MIR1520b^c^	GUGACAGUCAUCAUUUAAUAAGA	23	--
gma-MIR1520c^c^	UUCAAUAAGAACGUGACACGUGA	23	--
gma-MIR1520d^c^	AUCAGAACAUGACACGUGACAA	22	--
gma-MIR1521	CUGUUAAUGGAAAAUGUUGA	20	--
gma-MIR1522	UUUAUUGCUUAAAAUGAAAU	20	--
gma-MIR1523	AUGGGAUAAAUGUGAGCUCA	20	--
gma-MIR1524	CGAGUCCGAGGAAGGAACUCC	21	--
gma-MIR1525	UGGGUUAAUUAAGUUUUUAGU	21	--
gma-MIR1526	CCGGAAGAGGAAAAUUAAGCAA	22	--
gma-MIR1527	UAACUCAACCUUACAAAACC	20	--
gma-MIR1528	AUAGAUUAGAUCAAUAUAUUAGU	23	--
gma-MIR1529	UUAAAGGAAACAAUUAAUCGUUA	23	--
gma-MIR1530	UUUUCACAUAAAUUAAAAUAU	21	--
gma-MIR1531	UCGUCCAUAUGGGAAGACUUGUC	23	--
gma-MIR1532	AACACGCUAAGCGAGAGGAGCUC	23	--
gma-MIR1533	AUAAUAAAAAUAAUAAUGA	19	--
gma-MIR1534	UAUUUUGGGUAAAUAGUCAU	20	--
gma-MIR1535	CUUGUUUGUGGUGAUGUCU	19	--
gma-MIR1536	AAGCAGAGACAAAUGUGUUUA	21	
gma-MIR171b	CGAGCCGAAUCAAUAUCACUC	21	N (21 nt)
gma-MIR482	UUCCCAAUUCCGCCCAUUCCU	21	mi*

^a ^The most abundant read representing the family. Sequences with mismatches other than overhangs were marked as additional loci.

^b ^N denotes validation by Northern and the size of mature miRNA detected is indicated; mi* denotes validation by presence of miRNA* sequence in the library.

^c^The precursors of these miRNAs seems to be evolutionarily related, but the mature miRNAs are different from each other and we classified them as belonging to different families.

For about 55% of the conserved miRNA sequences in the set used to search for hairpin-forming precursors, we identified potential precursor sequences. All of these formed hairpin structures that fit the criteria for pri miRNAs (data not shown). The rest of the conserved miRNAs either lacked full-length cDNAs or corresponding precursors in the EST or WGS databases. Of the conserved miRNAs that we identified, 13 were from novel loci (representing 9 families) previously unidentified in soybean (Table 2). Over all, we identified 12 conserved miRNA families using the above approach. Of these, two families also had miRNA* sequences present in the library. As expected, we were able to validate several of the conserved miRNA families by Northern expression analysis (Table 2, see below). Thus, in addition to novel miRNAs, the study also revealed novel loci for conserved miRNAs from the soybean genome.

**Table 2:** Conserved miRNAs identified from soybean roots

**miRNA Identity**				
	
**Family**	**miRNA ID**^a^	**Sequence^b ^(5'-3')**	**Length**	**Detection method^c^**
156	gma-MIR156a	UGACAGAAGAGAGUGAGCAC	20	H, P
	gma-MIR156c,d,e	UUGACAGAAGAUAGAGAGCAC	21	H
	**gma-MIR156?**	UGACAGAAGAGAGAGAGCACA	21	H
159	gma-MIR159	UUUGGAUUGAAGGGAGCUCUA	21	H
	**gma-MIR159?**	UUUGGAUUGAAGGGAGCUA	19	H
	**gma-MIR159?**	AUUGGAUUGAAGGGAGCUC	19	H
	**gma-MIR159b**	AUUGGAGUGAAGGGAGCUCCA	21	H, P
	**gma-MIR159c**	AUUGGAGUGAAGGGAGCUCCG	21	H, P, N (21 nt)
160	gma-MIR160	UGCCUGGCUCCCUGUAUGCCA	21	H
	**gma-MIR160?**	UGCCUGGCUCCCUGAAUGCCA	21	H, N (21 nt)
**162**	**gma-MIR162**	UCGAUAAACCUCUGCAUCCA	20	H, P
**164**	**gma-MIR164**	UGGAGAAGCAGGGCACGUGCA	21	H, P, N (21 nt)
166	gma-MIR166a,b	UCGGACCAGGCUUCAUUCCCC	21	H
	**gma-MIR166?**	UCGGACCAGGCUUCAUUCCCG	21	H, N (21 nt)
167	gma-MIR167a,b	UGAAGCUGCCAGCAUGAUCUA	21	H, P
	**gma-MIR167c**	UGAAGCUGCCAGCAUGAUCUG	21	H, P^d^
	**gma-MIR167?**	UGAAGCUGCCAGCAUGAUCUU	21	H
168	gma-MIR168	UCGCUUGGUGCAGGUCGGGAA	21	H, N (21 nt)
169	gma-MIR169	CAGCCAAGGAUGACUUGCCGG	21	H, P, N (21 nt)
	**gma-MIR169b**	CAGCCAAGGAUGACUUGCCGA	21	H, P
	**gma-MIR169c**	AAGCCAAGGAUGACUUGCCGA	21	H, P
171	**gma-MIR171a**	UUGAGCCGUGCCAAUAUCACG	21	H, P
	**gma-MIR171?**	UUGAGCCGCGCCAAUAUCACU	21	H
	**gma-MIR171?**	UUGAGCCGCGUCAAUAUCUCA	21	H
	**gma-MIR171?**	CGAGCCGAAUCAAUAUCACUC	21	H
172	gma-MIR172a,b	AGAAUCUUGAUGAUGCUGCAU	21	H, P, N (21 nt)
319	gma-MIR319a,b	UUGGACUGAAGGGAGCUCCC	20	H, P
	**gma-MIR319?**	UUGGACUGAAAGGAGCUCCU	20	H
	**gma-MIR319c**	UGGACUGAAGGGAGCUCCUUC	21	H, P
**390**	**gma-MIR390a**	AAGCUCAGGAGGGAUAGCGCC	21	H, P, mi*
	**gma-MIR390b**	AAGCUCAGGAGGGAUAGCACC	21	H, P
**393**	**gma-MIR393**	UCCAAAGGGAUCGCAUUGAUC	21	H, P, N (21 nt)
396	gma-MIR396a	UUCCACAGCUUUCUUGAACUG	21	H, P, N (21 nt), mi*
	gma-MIR396b	UUCCACAGCUUUCUUGAACUU	21	H, P
**397**	**gma-MIR397?**	UCAUUGAGUGCAGCGUUGA	19	H
**482?**	**gma-MIR482?**	UCUUCCCUACACCUCCCAUACC	22	H
**845?**	**gma-MIR845?**	UCGACUCUGAUACCAAUUGUUG	22	H
**894?**	**gma-MIR894?**	UUCACGUCGGGUUCACCA	18	H
**1055?**	**gma-MIR1055?**	AUUUAGAGGGUGUUUUCCAGUGU	23	H

^a ^Novel soybean miRNA families or loci identified in this study are shown bold faced.

^b ^Consensus sequence obtained by aligning all reads with the family. Sequences with mismatches other than overhangs were marked as additional loci

^c^'H' denotes identification based on homology to conserved miRNA in miRbase, P denotes identification based on analysis of hairpin forming precursor, N denotes validation by Northern analysis and (n) indicates the estimated size of miRNA detected by Northern analysis. Note: Northern probes may not differentiate between members of the same family due to high sequence identity.

^d ^Tested by Northern, but not detected.

'?' followed by MIR number indicates that IDs were not yet assigned by miRbase due to the absence of precursor sequence.

### Identification of miRNA sequences based on homology to known miRNAs identified in other plant species

As mentioned previously, we searched our library against sequences in miRbase to identify conserved miRNAs. We identified 20 different families of conserved miRNA sequences in our library based on homology (Table 2) comprising a total of 39 loci. Ten of these families (12 loci) had been identified in soybean earlier by screening EST libraries for similarity to conserved miRNAs [28]. By constructing a small RNA library, we identified 10 additional families and 27 new loci representing conserved miRNAs in soybean (Table 2).

Using both of these methods above, we identified 55 families of miRNAs in soybean and of these 35 were novel. We also identified novel precursors and loci for conserved miRNAs. The identified miRNA sequences constituted about 3% of the total number of reads (Table 3). This number is low compared to other recent large-scale analyses of small RNA libraries [22,23]. The lower abundance in our library could be due to the fact that our library was constructed only from root tissue whereas the others were from whole plants or a combination of libraries from different tissues. In addition, our analysis excluded a large number of genuine miRNAs due to the lack of comprehensive genome sequence data.

**Table 3:** Composition of the small RNA library from soybean roots

**Type of small RNA**	**Number of reads**	**Frequency^a ^(%)**
miRNAs		
Conserved miRNAs	3758^b^	2.46
Novel miRNAs	793	0.52
(Total)	(4563)	(2.98)
Unidentified RNAs	38815	25.43
Chloroplast RNAs	832	0.55
Other small RNAs (rRNA, tRNA, snoRNA)	102,776	67.33
Fabaceae repeats	5,680	3.72

Total	152,654	100.00

^a ^Frequency is calculated as the percentage of RNAs in each class divided by the total number of RNAs that matched a known soybean sequence.

^b ^One conserved miRNA (ppt-miR896) was also identified in the search for other small RNAs

### Expression analysis and identification of B. japonicum-responsive miRNAs

We examined sequences in both libraries to identify differential abundance if any of the identified candidate miRNAs. Candidate miRNA sequences with at least 10 reads in both libraries combined were analyzed for abundance. As indicated in Table 4, there were several miRNA sequences that were up- or down-regulated in response to *B. japonicum *treatment in soybean roots. These included novel and conserved miRNAs.

**Table 4:** Relative abundance of selected miRNA reads in the control and Bj libraries^a^

	**Library**				
		
	**Control**		**Bj**		
		
**miRNA Family**	**Number of reads**	**Frequency^b^**	**Number of reads**	**Frquency^b^**	**Relative abundance (Bj/Control)**
171^d^	12	0.18	23	0.27	1.5
482	21	0.31	39	0.46	1.5
1507	22	0.33	38	0.44	1.4
390	12	0.18	19	0.22	1.3
160	39	0.58	60	0.70	1.2
1509	216	3.21	318	3.72	1.2
1508	28	0.42	40	0.47	1.1
396	54	0.80	77	0.90	1.1
159	584	8.68	831	9.73	1.1^c^
164	25	0.37	35	0.41	1.1
166	50	0.74	68	0.80	1.1
168	29	0.43	39	0.46	1.1
393	30	0.45	39	0.46	1.0
894?	7	0.10	9	0.11	1.0
1510	23	0.34	29	0.34	1.0
156	37	0.55	46	0.54	1.0
171	60	0.89	72	0.84	1.0
1511	11	0.16	13	0.15	0.9
167	52	0.77	61	0.71	0.9
1512	8	0.12	9	0.11	0.9
172	184	2.74	205	2.40	0.9
482?	20	0.30	22	0.26	0.9
169	31	0.46	31	0.36	0.8
162	6	0.09	6	0.07	0.8
319	50	0.74	45	0.53	0.7
1513	25	0.37	20	0.23	0.6

^a ^Only reads with a total abundance of ≥ 10 in both libraries combined are presented.

^b ^Frequency is calculated as the number of reads representing the family per thousand reads in the corresponding library that matched known soybean sequences. Number of reads in Control and Bj libraries that matched known soybean sequences were 67248 and 85406 respectively.

^c^The difference in read counts is statistically significant (P < 0.05) based on Poisson's distribution analysis.

^d ^The precursors of these miRNAs seems to be evolutionarily related, but the mature miRNAs are different from each other and we classified them as a novel miRNA.

**Figure 4:**
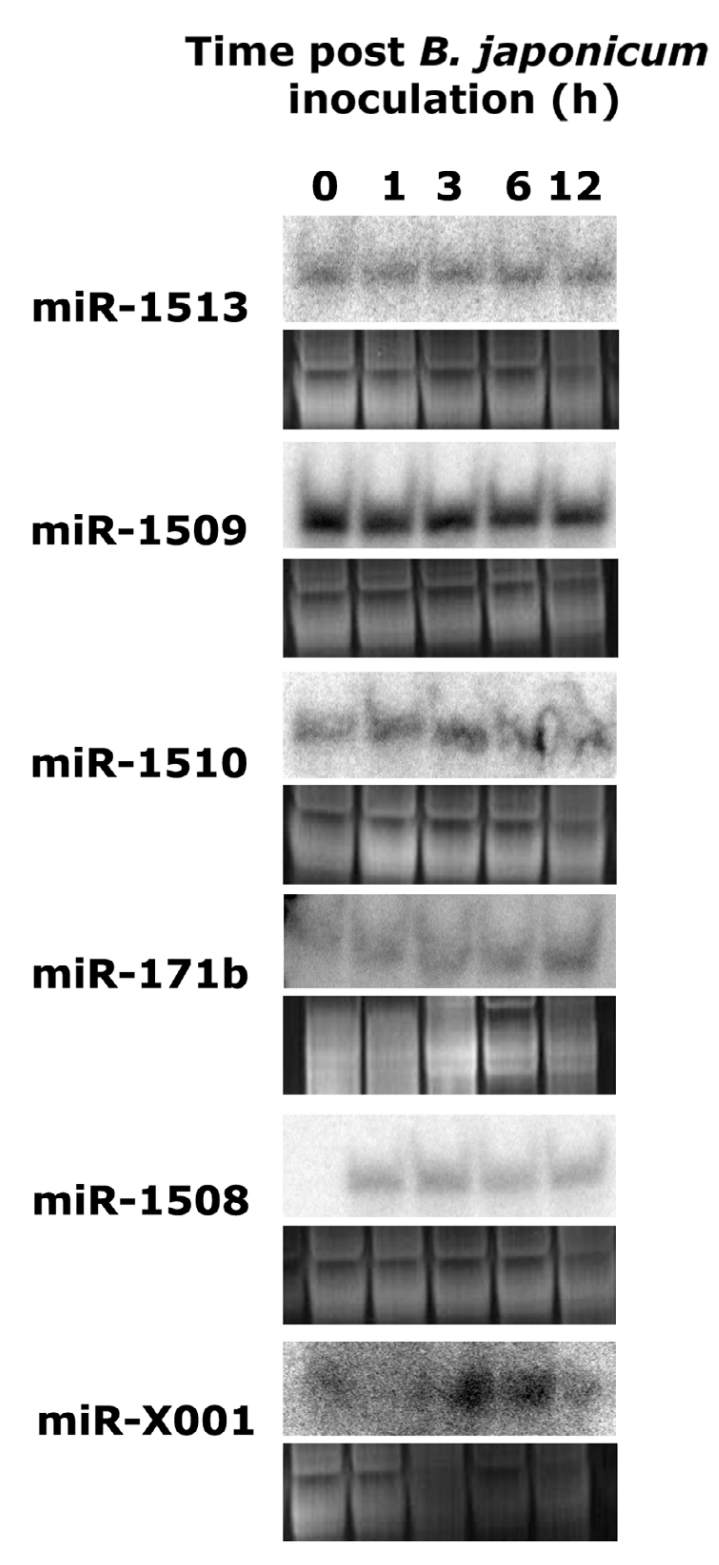
Expression of selected novel miRNAs in response to *B. japonicum *inoculation in soybean roots at different time points. Corresponding ethidium bromide stained gels show equal loading of total RNA in all lanes. (Note: The precursors of miR171 seem to be evolutionarily related, but the mature miRNAs are different from each other and we classified this as a novel miRNA).

**Figure 5:**
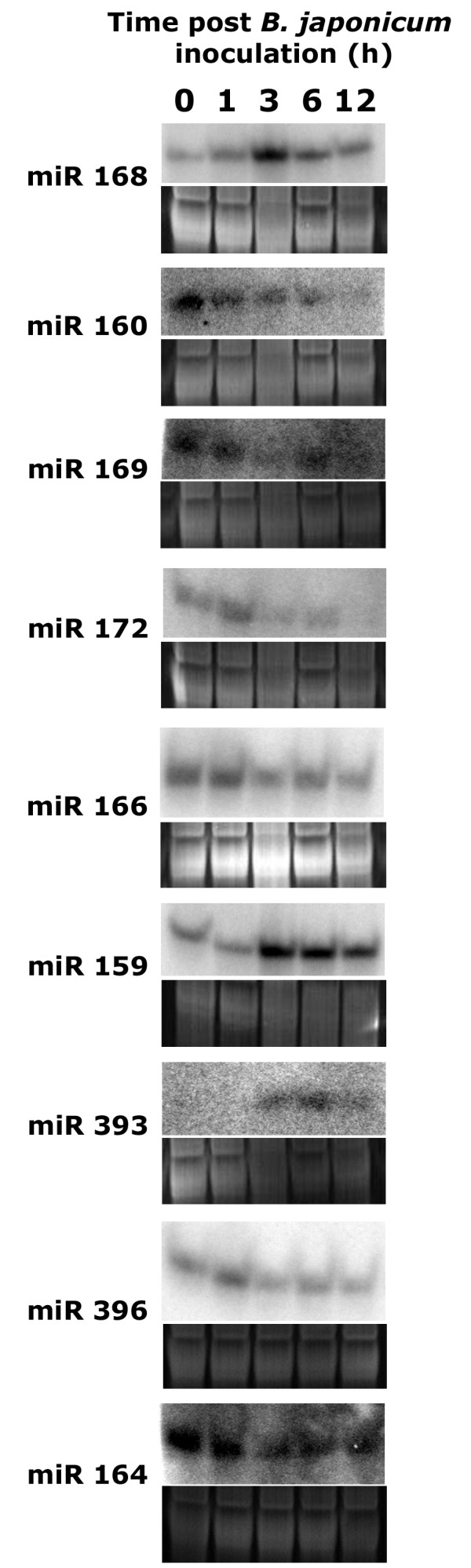
Expression of selected conserved miRNAs in response to *B. japonicum *inoculation in soybean roots at different time points. Corresponding ethidium bromide stained gels show equal loading of total RNA in all lanes.

We performed Northern analysis of selected miRNA candidates with two goals. 1. To experimentally validate these miRNAs and 2. To study the abundance of these miRNA during the early phases of nodulation. All of the 5 novel miRNAs tested were detectable by Northern expression analysis (Figure 4). Of the 10 conserved miRNAs tested, 9 were detectable by Northern expression analysis except miR167 (Figure 5). Thus, we were able to validate a majority of the identified miRNAs.

We performed a time course analysis of miRNA expression from 0 to 12 h after inoculation with *B. japonicum *cells. Interesting patterns of expression were observed for various miRNAs (Figures 4 and 5). A set of miRNAs tested were transiently up-regulated at 1 or 3 h after *B. japonicum *inoculation and were gradually down-regulated back to basal levels by 12 h (e.g. miR168, miR172). Significant up-regulation at 3 h post inoculation and sustained induction was observed for another set of miRNAs (e.g. miR159, miR393). Down-regulation of a set of miRNAs was also observed in response to *B. japonicum *(e.g. miR160, miR169). As expected, we also observed a number of miRNAs that remained unaffected by *B. japonicum *inoculation (e.g. miR1510, miR1509). Interestingly, we observed coordinated expression of certain miRNAs implicated in signal transduction of the plant hormone auxin (see Discussion).

### Identification and verification of potential miRNA targets

We sought to identify potential targets silenced by the identified miRNAs in soybean. Criteria and methods developed by Bartel, Carrington and Weigel labs [29-31] have enabled well-defined identification of potential targets for plant miRNAs with very few false-positives. We used the miRU online search utility [32] to identify predicted targets of candidate miRNAs and then examined the results manually to narrow down potential targets that fit the recently developed criteria [30,31]. The potential targets of both known and novel miRNAs were identified with varying levels of confidence. The results of the analysis are presented as supplementary data [see Additional file 1]. Some miRNA families had multiple targets, often functionally divergent with homologous sequences. In these targets, the putative miRNA binding-sites were often located in highly conserved regions.

**Figure 6:**
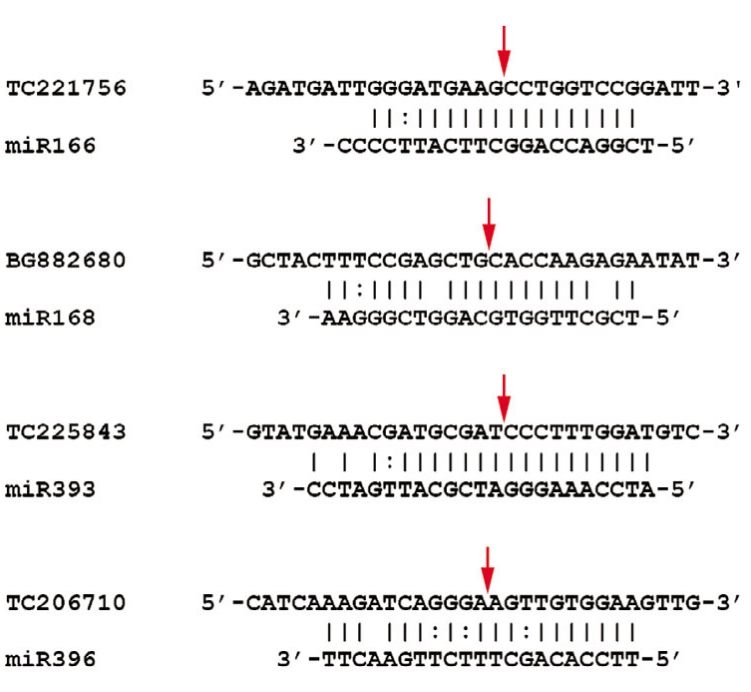
Cleavage sites of selected miRNA targets as identified by 5'-RACE analysis. For each miRNA, the target sequence is shown at the top and the miRNA sequence at the bottom. Perfectly complementary bases are shown connected by solid lines and G:U wobbles by dotted lines. The red arrows indicate the site of cleavage. Targets are labeled with TC or EST IDs assigned in The Gene Index [52].

For a subset of miRNAs, we experimentally verified the cleavage of selected targets by 5'-RACE analysis. We isolated polyA RNA from mock-inoculated and *B. japonicum*-inoculated (3 h) soybean roots and subjected the RNA to a 5'-RACE reaction (see Methods). We examined the presence of miRNA-directed cleavage products by PCR using primers specific to the target of interest. We were able to verify the cleavage of target miRNA for four of the identified miRNA families (Figure 6). The targets of miR166 and miR393 are well-conserved in other species. Consistently, we also observed cleavage of transcripts encoding a homeodomain protein [TGI:TC221756; 4 clones] and a TIR1-like protein [TGI:TC225843; 3 clones] by these miRNAs respectively. The identified targets of miR168 [TGI:BG882680; 1 clone] and miR396 [TGI:TC206710; 2 clones] in soybean seem to be non-conserved. These targets encode a putative protein kinase and a cysteine protease respectively. In all these cases, the site of cleavage corresponded to the position between the 10^th ^and 11^th ^nt of the miRNA.

## Discussion

We identified 55 candidate miRNA families from soybean roots. Of these 20 are conserved in other plant species and 35 are novel and some of these novel ones might be unique to soybean or legumes. We have also experimentally verified the expression of a selected set of known and novel miRNAs. The target mRNAs potentially regulated by these putative miRNAs were also identified using a bioinformatics approach and a subset of them experimentally validated by 5'-RACE analysis.

### Challenges in identifying miRNAs from species with little genome sequence information

Construction and high throughput sequencing of small RNA libraries seems to be the most efficient method for miRNA identification especially in species for which complete genome sequence information is not available. However, effective authentication of miRNAs is still best achieved by analyzing potential hairpin forming precursors identified from genomic or EST sequences. This approach identified the most number of novel miRNA sequences in our analysis (Table 1) and all 5 novel miRNAs tested were validated by Northern analysis (Figure 4). Based on our estimates using conserved miRNAs as internal control, this approach (analysis of hairpin forming precursors) identified about 49% of the conserved miRNAs in our library (Figure 1B). Failure to identify the rest of the conserved miRNAs was primarily due to non availability of either genome sequence data (~21%) or assembled genome shotgun sequences (~27%; Figure 1B). Analysis of our library using additional sequence data that might be available in the future [33] will lead to the identification of additional novel miRNAs. Another approach to the identification of miRNAs (without cloning) is based on similarity to previously known miRNAs in other species [28,34]. Obviously, this approach has the limitation of not being able to identify new or species-specific miRNA families. This approach has successfully identified 22 miRNAs from soybean ESTs. Another bioinformatics approach to identify novel miRNAs from species with little or no genome sequence information is to search for EST sequences with potential ability to form hairpin structures which has not been attempted in soybean to our knowledge.

We also identified conserved miRNAs in our library based on homology to known miRNAs listed in miRbase and this method is completely independent of the availability of genome sequence. This approach identified 20 families of miRNAs (Table 3) largely due to previous identification and characterization of miRNAs from other plant species that have their complete genome sequenced [15,17-19,22]. As expected, a number of conserved miRNAs were independently identified by the presence of potential hairpin forming precursor as well (Table 2).

Another approach to identify miRNAs in the absence of a genome sequence is to identify miRNA:miRNA* pairs in the small RNA sequence libraries themselves. We identified several conserved miRNAs using this approach and at least one novel miRNA which was validated by Northern expression analysis (miRX001; Figure 4). This approach may be promising for identifying regulatory RNAs in species with little or no genome sequence information. The most efficient method in identifying novel miRNAs seems to be the identification of potential hairpin forming precursors encompassing the mature miRNA sequence of interest (obtained by cloning) in either genomic or EST sequences. Combined use of these approaches might be the key to unravel novel miRNA loci and families in species with little or no genome sequence data.

### miRNA regulation during nodulation

In addition to the identification of miRNA sequences, we also examined the regulation of several known and novel miRNAs in response to *B. japonicum *inoculation in soybean roots. Thus far, only one miRNA has been shown to affect nodule development. The target of miR169, a CCAAT-binding transcription factor MtHAP2-1 has been shown to be critical for nodule development in *Medicago truncatula *[35]. Since miRNAs largely act as 'early' regulators of signal transduction by modulating the levels of transcription factors, we chose to construct the library from soybean roots 3 h post rhizobial inoculation. A number of gene expression changes have been observed at this time point in soybean roots [36]. We observed differential regulation of miRNAs that target a wide variety of target genes encoding transcription factors, receptor kinases, proteases, water channels and metabolic enzymes among others in response to *B. japonicum *inoculation [see Additional file 1]. The differential regulation of a subset of these miRNAs were also verified independently and in more detail by Northern analysis (Figures 4 and 5). In general, for about half of the miRNAs tested, the expression patterns observed by Northern analysis were consistent with the abundance of miRNA reads in the libraries. Discrepancies could be due to differences in cloning efficiency of certain miRNAs [21]. In addition, our analysis excluded a portion of the library due to unavailability of genome sequence data. This would also affect the abundance calculations due to missing family members/loci. In general, large-scale sequencing of small RNA libraries seems to be a valid method to study miRNA abundance.

Our study has provided several candidate miRNAs to test for their role in nodulation. For example, transient up regulation of miR172 (Figure 5) which regulates a putative Apetala2-like transcription factor, down-regulation of miR166 and miR396 (Figure 5) targeting an HD-ZIPIII-like transcription factor and a cysteine protease respectively are potentially novel regulatory elements during nodulation. We also identified down-regulation of miR169 (Figure 5) shown to play a role in nodulation earlier [35]. At least two putative miR169 targets predicted in soybean are highly identical (not shown) to the *M. truncatula *HAP2-1 functionally shown to play a role in nodulation. Functional analyses of these novel target genes in nodulation will yield interesting and useful information on their role in nodule signaling and development.

### miRNA regulation of auxin homeostasis/signaling

The plant hormone auxin regulates a number of developmental and physiological processes including nodulation. Several miRNA loci have been suggested to play a role in auxin homeostasis/signaling. For example, miR167 regulates the transcript levels of auxin response activator ARF8 [29,37] and miR160 regulates the transcript levels of auxin response repressors ARF10, 16 and 17 [38]. ARF8 and ARF17 regulate the transcription of GH3-like genes which encode auxin-amino acid conjugating enzymes that might regulate free auxin levels in the plant. ARF8 negatively regulates free auxin levels by upregulating these conjugating enzymes [39] whereas ARF17 downregulates them presumably increasing free auxin levels [40]. We observed down-regulation of an miR160 family member in response to *B. japonicum *suggesting that ARF17 levels increase and perhaps free auxin levels as well. ARF17 levels have also been shown to be regulated by ARGONAUTE1 (AGO1), a core component of the RISC complex [41]. Increased levels of ARF17 transcripts were observed in *ago1 *mutants of Arabidopsis [40]. We also observed increasing levels of an miR168 family member (presumably down regulating AGO1 levels) in response to *B. japonicum*. The opposite regulation of AGO1 and ARF17 levels in soybean roots is consistent with previous observations that AGO1 might negatively regulate ARF17 levels.

Other miRNAs suggested to play a role in auxin signaling include miR393 that regulates the auxin receptor TIR1 and miR164 that regulates the levels of a NAC1 transcription factor [29]. These miRNAs were up- and down-regulated respectively in response to *B. japonicum *inoculation in soybean roots. These observations suggest that endogenous regulatory RNAs might modulate auxin signaling and/or homeostasis during nodulation. While it still needs to be confirmed if the corresponding targets are silenced and what role they play in nodule signaling and development, these data provide an entry point for the study of miRNA regulation of early auxin signaling during nodulation.

## Conclusion

Cloning and sequencing of ~350000 small RNAs from *B. japonicum*-inoculated soybean roots and further bioinformatics analyses identified 55 families of miRNAs of which 35 were novel. We estimated that our study identified ~50% of the miRNAs in soybean genome. The availability of complete soybean genome sequence and its assembly would enable the identification of most of the remaining miRNAs. We also identified a number of miRNAs differentially regulated by *B. japonicum *inoculation. Results from the study might help advance our understanding of legume-rhizobia symbiosis.

## Methods

### Plant material and B. japonicum treatment

Soybean (*Glycine max *cv. Williams82) seeds were surface sterilized by treating with 8% Clorox for 4 min followed by 70% ethanol for 4 min. The seeds were then rinsed three times with sterile deionized water. Surface sterilized seeds were planted in a 4" pot filled with 1:3 (v/v) mixture of sterilized vermiculite and perlite (Hummer International, St Louis, MO) and watered with nitrogen free plant nutrient solution (N^- ^PNS). *B. japonicum *cells (USDA110; a kind gift from Dr. Gary Stacey, University of Missouri, Columbia, MO) were grown in Vincent's rich medium [42] and resuspended in N^-^PNS to a final concentration of 10^8 ^cells ml^-1 ^(OD_600 _= 0.08; [43]). One-week-old seedlings were flood-inoculated with the above *B. japonicum *cell suspension (15 ml/pot). For mock-inoculation, seedlings were watered with N^-^PNS. After 3 h of treatment, seedlings were uprooted, rinsed briefly in sterile deionized water to remove vermiculite/perlite particles, immediately frozen in liquid N_2 _and stored at -80°C.

### Small RNA isolation, library construction and sequencing

Small RNA isolation and library construction was performed as described by [19] except that the PCR products were directly sequenced instead of cloning in to a plasmid vector. RNA molecules corresponding to 15 – 30 nt in size were isolated from mock-inoculated and *B. japonicum*-inoculated roots and adapters ligated to the 3' and 5' ends sequentially as previously described [19,44]. These molecules were subjected to a cDNA synthesis reaction followed by PCR amplification [19]. One sequencing run was performed on 3 μg of nucleic acid from each library by 454 Life sciences [45] resulting in a total of 354,000 small RNA reads (Figure 1A).

### Bioinformatic analyses and identification of candidate miRNAs

The adapter sequences (5'-AAACCATGGTACTAATACGACTCACTAAA-3' and 5'-TTTTCTGCAGAAGGATGCGGTTAAA-3') and low quality regions were subsequently removed using Lucy [46] with default settings except two parameters (-minimum 16 and -threshod 90). In addition, contaminants were removed using Seqclean [47] based on GenBank's Univec database [48]. These sequences were searched against soybean WGS traces (~700,000 reads) downloaded from the National Center for Biotechnology Information (NCBI) trace archive ([49];9/21/2006), draft genomes of *Medicago truncatula *([50];9/25/06) and *Lotus japonicus *([51];9/21/06) and TIGR soybean Gene Index ([52];Ver. 12) using MegaBLAST to identify sequences with perfect matches to at least one sequence in these databases. The resulting ~153000 sequences were searched for perfect sequence matches to non-regulatory RNA sequences in the following databases using MegaBLAST, to exclude non-regulatory RNA sequences: soybean rRNA sequences in GenBank, plant large subunit-rRNAs [53], Arabidopsis rRNA, tRNA, snoRNA sequences [54], TIGR Fabaceae_repeats (Ver. 2), and soybean chloroplast genome (NC_007942). For elimination of 'orphan' sequences, we searched for reads that were represented only once in both libraries combined. Reads that had a perfect match to another read except for overhangs were considered to represent the same sequence. A total of 26722 "non-orphan" sequences (5872 unique reads) remained for further analysis. After excluding reads that had a match to more than 20 WGS reads, we retained 18868 reads (4959 unique reads).

### Identification of conserved miRNAs

For the identification of conserved miRNAs, library sequences were searched against plant miRBase ([55];Version 10), which contained 1284 mature miRNA/miRNA* and 1220 corresponding precursor sequences with the BLASTN parameters: -W 7 -S 1 -F F, bit scores > 30. These sequences were then clustered to generate miRNA families. miRbase had eleven soybean miRNA families listed and all of them were identified via computational approaches [34,56].

### Prediction of potential pri miRNA sequences

The genomic and cDNA sequences that had a region with perfect sequence match to at least one of the 4959 unique sequences from above were used for novel miRNA discovery. Those redundant soybean WGS sequences hit by small RNA sequences were assembled using CAP3 [56]. The consensus sequences with other cDNA sequences together were aligned with each given small RNA to search for its complementary sites (i.e. potential corresponding miRNA/miRNA*) within upstream or downstream 500-bp window using WU-BLAST (gapped WU-BLASTN parameters: M = 1 N = -1 S = 10 W = 6 Q = 1 R = 1). The candidate pri miRNA sequences were folded using MFOLD [57] and the resulting structures were manually examined for the criteria proposed by [23] i.e. to be designated as candidate miRNA, a 25 bp region of the stem centered around the mature miRNA should have less than 8 unpaired nucleotides (together on both arms) and no more than three consecutive unpaired nucleotides, of which no more than two were asymmetrically bulged.

### miRNA expression analysis and target validation

RNA isolation and Northern analysis were performed essentially as described by [31]. Total RNA was isolated from soybean roots inoculated with *B. japonicum *cells at different time points (0, 1, 3, 6 and 12 h) using Trizol (Invitrogen, Carlsbad, CA) reagent. Ten μg of total RNA was separated on a Criterion 15% TBE-Urea gel (Bio-Rad Labs, Hercules, CA), transferred to positively charged nylon membranes (Roche, Indianapolis, IN) using a Criterion wet transfer blot apparatus (Bio-Rad Labs, Hercules, CA) and fixed on the membranes using an UV cross-linker (Stratagene, La Jolla, CA). Oligonucleotide probes (IDT Inc., Coralville, IA) complementary to miRNAs were end-labeled with γ-^32^P ATP (Perkin Elmer, Shelton, CT) using Optikinase (USB Corporation, Cleveland, OH) and purified using a nucleotide removal column (Qiagen, Valencia, CA). RNA blots were pre-hybridized for at least 1 h with Perfect Hybplus (Sigma, St Louis, MO) hybridization buffer and hybridized with the labeled probes for 16–24 h at 38°C. Blots were then washed twice (10 min ea) with 2× SSC 0.2% SDS at 42°C, exposed to phosphorimager screens (Amersham, Piscataway, NJ) for 1 h and imaged using a Typhoon phosphorimage analyzer. Blots with a higher background were additionally washed once at 50°C and re-exposed.

For experimental validation of target cleavage, total RNA was extracted from soybean roots at 0 and 3h after *B. japonicum *inoculation. PolyA RNA was isolated from these preparations using polyAtract mRNA isolation system III (Promega, Madison, WI). PolyA RNA was subjected to a 5'-RACE reaction using Gene Racer core kit (Invitrogen, Carlsbad, CA) omitting calf intestinal phosphatase and tobacco acid pyrophosphatase treatments. Gene-specific reverse primers were designed from targets to be verified and used in combination with the generacer 5' primer to amplify cleaved transcripts. PCR products were cloned in to pCR4 TOPO TA vector (Invitrogen, Carlsbad, CA) and sequenced (Cogenics, Houston, TX).

## List of abbreviations

AGO, ARGONAUTE; ARF, auxin response factor; DCL, DICER-like; EST, expressed sequence tags; LCO, lipochitooligosaccharide; miRNAs, microRNAs; N^-^PNS, nitrogen free plant nutrient solution; pri miRNA, primary microRNA; RACE, random amplification of cDNA ends; WGS, whole genome shotgun.

## Authors' contributions

SS and OY conceived, designed and coordinated the study, J-KZ and WBB participated in the design and coordination of the study, RS constructed the small RNA library, YF and WBB performed bioinformatics analyses, SS performed small RNA and target analyses. All authors read and approved the final manuscript.

## Supplementary Material

Additional file 1 Potential targets of soybean miRNAs identified in the study. A table listing the potential targets of miRNAs identified in this study. Click here for file
