# Supplementary material for: Novel and nodulation-regulated microRNAs in soybean roots
Source: BMC Genomics. 2008 Apr 10;9:160. doi: 10.1186/1471-2164-9-160 (PMC2335117; doi:10.1186/1471-2164-9-160)
Supplement: Additional file 1 — Potential targets of soybean miRNAs identified in the study. A table listing the potential targets of miRNAs identified in this study. [file 1471-2164-9-160-S1.pdf]

Supplementary table

Potential targets of soybean miRNAs identified in the study.

miRNA family[a]	Target[b]	Score[c]
miR169	TC233387 RAPB protein<br>TC220009 HAP-like transcription factor<br>BQ611496	2.5<br>3.0<br>3.0
miR166	BM309730 Homeodomain transcription factor<br>TC221756 Class III homeodomain Zip transcription factor<br>TC230399 Class III homeodomain Zip transcription factor	3.0<br>3.0<br>3.0
miR396	TC231667 annotation not available<br>TC223232 annotation not available	3.0<br>3.5
miR569	TC225608 Resistance protein R8<br>TC225611 Resistance protein R1<br>TC225617 Resistance protein R3	3.0<br>3.0<br>3.0
miR172	BI320499 putative transcription factor<br>TC205405 HAP2B like transcription factor<br>TC208557 annotation not available	1.5<br>2.0<br>2.0
miR164	TC216430 NAC1 domain protein<br>TC218305 NAM-like protein<br>TC221831 NAM-like protein	2.0<br>3.5<br>3.5
miR159	TC233211 ARF-like protein<br>CO984960 annotation not available	3.0<br>4.0
miR168	BG882680 S/T protein kinase	3.5
miR160	TC213894 annotation not available<br>TC208983 ARF10-like protein	0.5<br>1.0
miR156	TC209333 Squamosa promoter binding-like protein<br>TC210466 CBS domain protein	0.0<br>0.0
miR167	CD411229 Annotation not available<br>TC221608 annotation not available	0.0<br>0.5
miR171	CO979466 annotation not available<br>TC217781 Scarecrow-like protein	2.0<br>2.5
miR319	CO984960 annotation not available	0.0
miR393	TC225844 Transport inhibitor response-like protein<br>TC203511 Transport inhibitor response-like protein<br>TC225845 Transport inhibitor response-like protein	2.0<br>2.5<br>2.5
miR-1514	TC208997 annotation not available<br>TC219451 annotation not available<br>TC223409 Plasticity related 2a-like gene	1.0<br>1.0<br>2.0
miR-1508	AW306720 Syringolide-induced protein<br>TC204652 Histone H1<br>TC207519 annotation not available	4.0<br>4.0<br>3.5
miR-1510	TC207788 Resistance protein KR1<br>AW620306 Resistance protein<br>TC223320 Potential resistance protein	3.5<br>3.0<br>4.0

miR-1513	BG790677 annotation not available<br>TC232958 annotation not available	3.0<br>4.0
miR-1507	TC219096 Mycolyl transferase-like protein<br>TC218389 Putative nitrate transporter<br>TC221480 annotation not available	3.0<br>3.5<br>4.0
miR-1509	BI785214 annotation not available	2.0
miR-1512	TC206050 annotation not available<br>TC213545 Myosin-like protein<br>TC217383 Copine-I like protein	3.0<br>3.5<br>3.5
miR-1515	TC222568 annotation not available	4.0
miR-1516	TC224565 Glyceraldehyde-3-phosphate dehydrogenase<br>TC211509	3.5<br>4.5
miR-1518	TC226145 similar to S/T protein kinase<br>TC220660 annotation not available	3.5<br>3.0
miR-1519	TC231879 annotation not available	3.0
miR-1536	TC210530 annotation not available	3.5
miR-1524	BM308243 annotation not available	3.5
miR-1525	AW704761 annotation not available<br>BI699330 annotation not available<br>BU084298 annotation not available	1.0<br>2.0<br>2.0
miR-1530	TC213778 annotation not available	2.0

[a] Families for which no target with a score of ≥ 4.0 are not listed
[b] Target sequences were identified using the miRU on-line utility (Zhang 2005) and manually scored. Sequences are denoted by TC or EST IDs and the annotation from soybean gene index (http://compbio.dfci.harvard.edu/tgi/cgi-bin/tgi/gimain.pl?gudb=soybean).
[c] Score is based on criteria developed by Allen et al. (2005). In addition, targets that had a mismatch in the 10th or 11th nt of miRNA were also excluded (Schwab et al. 2005).

References:

Allen, E., Z. Xie, A.M. Gustafson, and J.C. Carrington. 2005. microRNA-directed phasing during trans-acting siRNA biogenesis in plants. *Cell* **121:** 207-221.
Schwab, R., J.F. Palatnik, M. Riester, C. Schommer, M. Schmid, and D. Weigel. 2005. Specific effects of microRNAs on the plant transcriptome. *Dev Cell* **8:** 517-527.
Zhang, Y. 2005. miRU: an automated plant miRNA target prediction server. *Nucleic Acids Res* **33:** W701-704.
